# Age-related High-frequency Hearing Loss Is Not Associated With Horizontal Semicircular Canal Function

**DOI:** 10.1097/AUD.0000000000001252

**Published:** 2022-10-18

**Authors:** Nick M. A. Schubert, Catelijne G. Roelofs, Rolien H. Free, J. Esther C. Wiersinga-Post, Sonja J. Pyott

**Affiliations:** 1 Department of Otorhinolaryngology and Head/Neck Surgery, University of Groningen, University Medical Center Groningen, Groningen, The Netherlands; 2 Graduate School of Medical Sciences Research School of Behavioural and Cognitive Neurosciences, University of Groningen, Groningen, The Netherlands.

**Keywords:** Cerebellopontine angle tumors, Hearing loss, Presbyacusis, Presbyequilibrium, Vestibular dysfunction

## Abstract

Investigate the association between age-related hearing loss and reduced peripheral vestibular function using paired assessments of high-frequency hearing and horizontal semicircular canal (HSC) function. We hypothesized that age-related high-frequency hearing loss would be correlated with reduced HSC function and, therefore, useful to predict age-related vestibular hypofunction.

**Design:**

We conducted a single center, retrospective cross-sectional study in a tertiary/academic referral hospital. This study included 185 patients who were diagnosed with a cerebellopontine angle (CPA) tumor and referred to the academic hospital to evaluate treatment options. Data collected included pure-tone audiometry, caloric reflex test, video head-impulse test (vHIT), and medical history. High-frequency hearing loss was quantified by the high Fletcher index (hFI), and horizontal semicircular canal (HSC) function were quantified by the caloric reflex test and vHIT.

**Results:**

We observed a significant association between age and high-frequency hearing loss that was significantly worse in men compared with women. In contrast, we observed no significant association between age and HSC function assessed by either the caloric reflex test or vHIT. We observed associations between HSC function and sex, with male sex predicting reduced HSC function by caloric reflex testing but enhanced HSC function by vHIT. High-frequency hearing loss did not predict HSC hypofunction.

**Conclusions:**

We found no evidence indicating age-related decline in HSC function or an association between age-related high-frequency hearing loss and age-related decline in HSC function. We did observe sex-specific differences in HSC function. Our study highlights the need for sex-specific normative values for identifying age-related reduced peripheral vestibular function and for future work linking comprehensive assessments of inner ear function with tests of balance and stability to understand the complex interactions underlying hearing loss and imbalance, especially in the elderly.

## INTRODUCTION

The inner ear is responsible for the sense of hearing and balance. Loss of non-regenerating sensory hair cells and primary auditory and vestibular neurons is the most common cause of age-related hearing loss (Wu et al. 2020) and reduced peripheral vestibular function (Ji & Zhai 2018) resulting in imbalance, dizziness, and visual blurring. Shared molecular mechanisms (Paplou et al. 2021) may underlie sensorineural loss in both the cochlea, the auditory portion of the inner ear, and the vestibule, which includes the three semicircular canals—the horizontal, superior, and posterior semicircular canals that are sensitive to angular accelerations (rotations)—and two otolith organs—the saccule and utricle that are sensitive to linear (straight line) accelerations. It is important to note that, age-related loss of cochlear and vestibular function are associated with reduced quality of life, social isolation, and increased risk of cognitive decline (Agrawal et al. 2018; Loughrey et al. 2018; Dobbels et al. 2019; Dixon et al. 2020). Furthermore, both hearing loss and dizziness are independently associated with a greater risk of falls, the most common cause of traumatic injury and death in the elderly (Herdman et al. 2000; Lin & Ferrucci 2012). When clinically assessed, age-related hearing loss and reduced peripheral vestibular function are both quite prevalent: more than half of adults over the age of 70 years have hearing impairment (Lin 2011) or balance dysfunction (Agrawal et al. 2009). Better understanding of the association between age-related hearing loss and reduced peripheral vestibular function would improve screening approaches that aim to increase detection of vestibular deficits, which can go unnoticed, and inform strategies to reduce falls and improve healthy aging.

Age-related hearing loss is commonly assessed using pure-tone audiometry (PTA) and shows a stereotyped pattern of high-frequency hearing loss that worsens and extends to lower frequencies with age (Parthasarathy et al. 2020). Comprehensive investigation of the five vestibular end-organs in 50 adults over the age of 70 years revealed declining vestibular function across all end-organs but differences in the extent of decline among the end-organs (Agrawal et al. 2012). Specifically, horizontal, superior, and posterior canal hypofunction were highly concurrent within individuals and most prevalent (≈90% of individuals, revealed by head thrust dynamic visual acuity testing: htDVA), followed by saccular hypofunction function (≈60%, revealed by cervical vestibular-evoked myogenic potentials: VEMPs), and utricular hypofunction (≈20%, revealed by ocular vestibular-evoked myogenic potentials: oVEMPs). These findings are consistent with previous assessments of age-related decline in specific vestibule end-organs (Baloh et al. 2001; Zapala et al. 2008; Maes et al. 2010; Ji & Zhai 2018) and histological assessments indicating more prevalent (Rosenhall 1973) and greater (Rosenhall & Rubin 1975; Walther & Westhofen 2007; Merchant et al. 2000; Zalewski 2015; Ji & Zhai 2018) sensory hair cell loss in the cristae ampullares of the semicircular canals relative to the otolithic maculae. When subsequently investigating the association between age-related hearing loss, assessed using PTA, with vestibular end-organ hypofunction in this same group, a significant correlation between high-frequency hearing loss and reduced function of the saccule but not utricle or semicircular canals was found (Zuniga et al. 2012).

These findings, increased prevalence of semicircular canal compared with otolithic hypofunction among the elderly (Agrawal et al. 2012) and yet a lack of correlation between age-related hearing loss and age-related semicircular canal hypofunction (Zuniga et al. 2012) seemed at odds and prompted us to revisit the association between age-related hearing loss and reduced peripheral vestibular function using an expanded patient group and alternative assessments of semicircular canal function. The previous studies (Agrawal et al. 2012; Zuniga et al. 2012) assessed semicircular canal function using htDVA, which measures visual stabilization performance that depends not only on the vestibulo-ocular reflex (VOR) but also on other processes, including oculomotor function and cognitive processing, that may additionally be impaired by aging. Indeed, another study investigating age-related changes in semicircular canal function using an alternative method to probe horizontal canal function—the video head-impulse testing (vHIT), a computerized version of the head-impulse test (HIT) (Halmagyi & Curthoys 1988)—found that VOR was largely unaffected by aging even into the ninth decade of life (McGarvie et al. 2015). This study, however, did not have paired audiometric data to investigate potentially informative correlated changes in age-related hearing loss and vestibular hypofunction.

Therefore, in this study, we assessed semicircular canal, and specifically horizontal semicircular canal (HSC), hypofunction using two distinct but complementary methods: vHIT and caloric reflex testing. vHIT was used to probe HSC function at high frequencies (Alhabib & Saliba 2017). Caloric reflex testing, the most accepted method of evaluating peripheral vestibular function, was used to probe HSC canal at low frequencies (Perez & Rama-Lopez 2003). These measures of HSC function were correlated with paired measures of age-related high-frequency hearing loss, assessed using PTA, to investigate the association between age-related hearing loss and vestibular hypofunction. We hypothesized that age-related high-frequency hearing loss can be correlated with HSC function and to predict age-related HSC dysfunction.

## MATERIALS AND METHODS

### Study Design, Population, and Inclusion/Exclusion Criteria

A retrospective cross-sectional study was performed using patients that underwent routine diagnostic workup for cerebellopontine angle (CPA) tumors at a tertiary/academic referral hospital, between 2014 and 2021. At the time of referral, patients were already diagnosed with a CPA tumor and were referred to a tertiary hospital to evaluate treatment options. Patients underwent bilateral audiometric and vestibular assessment. Data from the unaffected ear were used to assess age-related hearing loss and reduced peripheral vestibular function and the association between hearing function and vestibular function. Patients reported no severe vestibular complaints. Exclusion criteria were: age <18 years, bilateral CPA tumors, and comorbidities that affect auditory or vestibular function, including history of ear surgery and labyrinthitis. Patients with reported cognitive deficits were also excluded. This study was approved by the Medical Ethical Review Board of the University Medical Center Groningen (UMCG) (number 2020/567).

### Data Collection

Audiometric, vestibular, and demographic data were collected from audiometric and vestibular testing datasets assembled by the Department of Otorhinolaryngology of the UMCG. All audiometric and vestibular assessments were conducted by audiologists from the hospital. Comorbidities and side of the CPA tumor were extracted from the electronic medical records. Information was collected for cardiovascular risk factors (hypertension, diabetes mellitus, heart failure, peripheral vascular disease, transient ischemic attack, cerebrovascular accident, myocardial infarction, and smoking), cognitive decline, and neurological disorders.

### Auditory Function Assessment

Auditory function was assessed using PTA performed in a soundproof room. Instrumentation included an audio-meter with headphones. Equipment was calibrated according to the International Standard Specifications for Audiometers (ANSI S3.6-1969). The test environment met the criteria for background noise in audiometric rooms as specified by the International Standard Criteria for Permissible Ambient Noise during Audiometric Testing (ISO 8253). Air conduction thresholds were obtained over the range of 250 to 8000 Hz, with increasing intensities from 10 to 120 dB HL. Outcome measures were summarized using the high Fletcher Index (hFI), which is the average threshold over 1000, 2000, and 4000 Hz. The hFI is well correlated with speech reception thresholds (Fletcher 1950).

### Vestibular Function Assessment

HSC function was assessed using caloric reflex testing and vHIT. For bilateral caloric reflex testing, the patient was placed supine with their heads lifted 30° to place the HSC in the vertical position. Each external auditory canal was alternately irrigated with a constant flow of cold (30°C) and warm (44°C) water for ≈40 seconds to induce caloric nystagmus. Nystagmus was recorded using infrared videonystagmography (Interacoustics, VNG VO425). The sum of the maximum slow phase velocity (SPV_max_, in °/s) at 30°C and 44°C after irrigation of the healthy ear was used as the outcome measure. For vHIT, the patient’s head was rapidly (velocity between 150 and 250 °/s) rotated (10° to 20°) in the horizontal plane. Compensating eye movements were measured using videography (Interacoustics EyeSeeCam). The VOR gain measured at 60 ms (Gain_60 ms_) when rotating the head toward the healthy side was used as the outcome measure. Because vHIT was not implemented until 2016 (2 years after caloric reflex testing was implemented) and because patients with neck problems did not undergo vHIT, not all patients included in this study underwent assessment by both caloric reflex testing and vHIT.

### Statistical Analysis

Following data collection, data were imported and processed in R (4.0.4) (Team & others 2013) and RStudio (1.4.1106), using the following packages: tidyverse (Wickham et al. 2019) and ggplot2 (Wickham 2016). Univariate analysis was performed with the compareGroup package (4.5.1) (Subirana et al. 2014). For normally distributed variables, means and standard deviations (SD) were calculated and presented as the mean with ± SD in parentheses. For categorical data, absolute numbers are presented with percentages in parentheses. To compare univariate differences between sexes, the following tests were used: Student’s *t* test for normally distributed variables, Kruskall-Wallis test for non-normally distributed variables, and the Fisher’s exact test for categorical variables. To compare auditory and vestibular function, Pearson’s correlation coefficient (r) was calculated. Finally, multivariable linear regression analyses were performed to identify associations between auditory and vestibular performance. Outcome measures (SPV_max_ or Gain_60 ms_) were used as continuous outcome variables, models were corrected for age and sex, and a *P* < 0.05 was considered statistically significant.

## RESULTS

### Background and General Characteristics of the Cohort

Paired audiometric and vestibular assessment were obtained from patients with unilateral cerebellopontine angle (CPA) tumors as part of the routine diagnostic workup. Thus, the paired audiometric and vestibular assessments obtained for this cohort afforded the excellent opportunity to examine the association of age-related hearing loss and vestibular function (in the healthy ear) of otherwise healthy, normally aging individuals. This cohort contained a total of 185 CPA tumor patients (56.7% female). Caloric reflex testing results were available for 177 patients, and vHIT results were available for 96 patients. Patients ranged in age from 24 to 85 years old (mean age of 62.3 ± 12.6 years), with no significant difference in age between males and females (Table 1). The number of comorbidities was relatively low for all patients and equivalent between sexes, with the exception that males showed significantly more cases of diabetes (11.2%) compared with females (2.86%, *P* = 0.046; Table 1).

**TABLE 1.** Descriptive characteristics of study population

	All	M	F	*P*	N
	N = 185	N = 80	N = 105		
Age (y)	62.3 (12.6)	63.0 (12.0)	61.7 (13.1)	0.472	185
Fletcher Index (dB HL)	24.1 (14.8)	26.8 (16.2)	22.0 (13.4)	0.034^*^	185
SPV (°/s)	49.2 (24.1)	42.3 (17.9)	54.6 (26.8)	0.001^*^	177
Gain @ 60ms	0.94 (0.23)	0.98 (0.16)	0.90 (0.27)	<0.001^*^	96
Healthy ear				0.07^*^	185
L	104 (56.2%)	44 (55.0%)	60 (57.1%)		
R	81 (43.8%)	36 (45.0%)	45 (42.9%)		
CPA tumor type				0.001^*^	185
Cavernoma	1 (0.54%)	0 (0.00%)	1 (0.95%)		
Cholesterol granuloma	1 (0.54%)	1 (1.25%)	0 (0.00%)		
Not specified CPA	16 (8.65%)	11 (13.8%)	5 (4.76%)		
Glomus jugulotympanicum paraganglioma	2 (1.08%)	0 (0.00%)	2 (1.90%)		
Hemangioblastoma	1 (0.54%)	1 (1.25%)	0 (0.00%)		
Meningeoma	10 (5.41%)	0 (0.00%)	10 (9.52%)		
Vestibular schwannoma	154 (83.2%)	67 (83.8%)	87 (82.9%)		
Hypertension	24 (13.0%)	9 (11.2%)	15 (14.3%)	0.698	185
TIA or CVA	6 (3.24%)	4 (5.00%)	2 (1.90%)	0.405	185
Peripheral vascular disease	1 (0.54%)	0 (0.00%)	1 (0.95%)	1	185
Heart failure	1 (0.54%)	1 (1.25%)	0 (0.00%)	0.432	185
Myocardial infarction	3 (1.62%)	2 (2.50%)	1 (0.95%)	0.579	185
Diabetes mellitus	12 (6.49%)	9 (11.2%)	3 (2.86%)	0.046^*^	185
Smoking (current)	3 (1.62%)	0 (0.00%)	3 (2.86%)	0.26	185
Smoking (former)	5 (2.70%)	3 (3.75%)	2 (1.90%)	0.654	185
Neurological	4 (2.16%)	0 (0.00%)	4 (3.81%)	0.135	185

Values for continuous parameters are presented as means with standard deviations between parentheses. Categorical data are presented as absolute values with percentages between parentheses.

* Indicates significant differences (*P* < 0.05).

CPA indicates cerebellopontine angle; CVA, cerebrovascular accident; F, females; M, males; TIA, transient ischemic attack

### Age-related Changes in Hearing

**Fig. 1.**
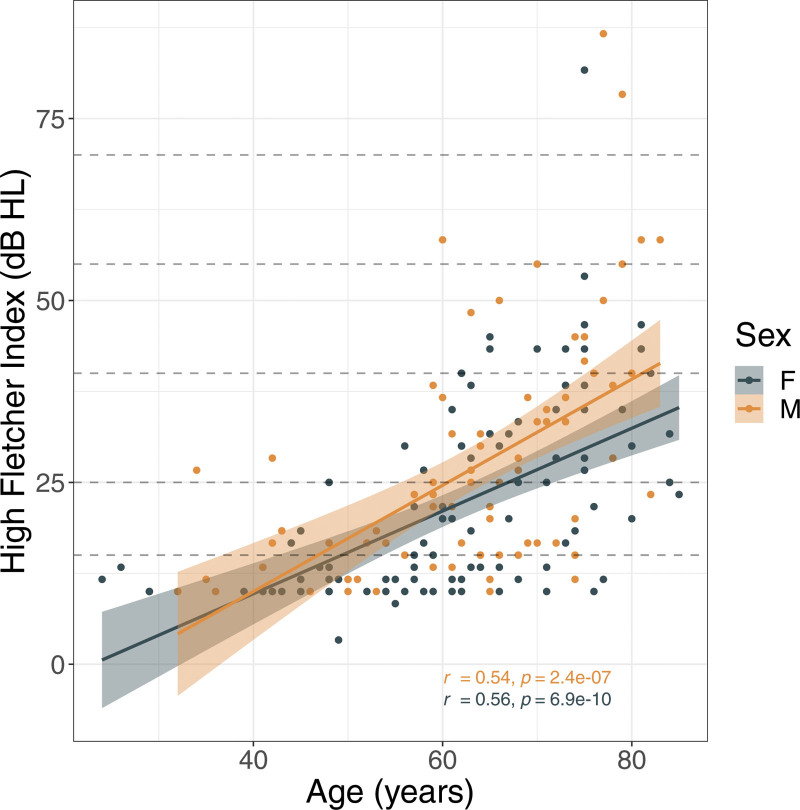
Positive correlation between age and hFI observed for both sexes. hFI is plotted as a function of age for males (orange, r = 0.54, *P* < 0.001) and females (blue, r = 0.56, *P* < 0.001). The dashed lines represent the different degrees of hearing loss ranging from slight (15 to 25 dB HL) to severe (>70 dB HL) (Clark 1981). hFI indicates high Fletcher Index.

Audiometric assessment was performed using PTA and quantified as the hFI as described in the Methods. The mean hFI across 185 patients was 24.1 ± 14.8 dB HL. Figure 1 shows that most of the patients fall in the normal to moderate hearing loss categories. The hFI was significantly greater in males (26.8 ± 16.3 dB HL) compared with females (22.0 ± 13.4 dB HL, *P* = 0.034; Table 1). A positive correlation between age and hFI was observed for both sexes (combined: r = 0.54, *P* < 0.001; males: r = 0.54, *P* < 0.001; females: r = 0.56, *P* < 0.001; Fig. 1).

### Age-related Changes in Vestibular Function

**Fig. 2.**
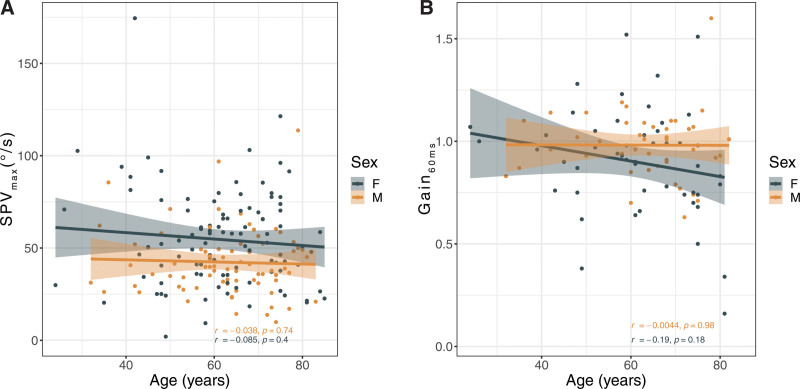
No correlation between age and horizontal semicircular canal function assessed using either caloric reflex testing (A) or vHIT (B). A, SPV_max_ (°/s) is plotted as a function of age for males (orange, r = –0.038, *P* = 0.74) and females (blue, r = –0.085, *P* = 0.4). B, Gain_60 ms_ is plotted as a function of age for males (orange, r = 0.004, *P* = 0.98) and females (blue, r = –0.19, *P* = 0.18).

Age-related change in vestibular function was assessed using two methods: caloric reflex testing and vHIT as described in the Methods. For caloric reflex testing, the mean SPV_max_ across 177 patients was 49.2 ± 24.1°/s and significantly reduced in males (42.3 ± 17.9°/s) compared with females (54.6 ± 26.8°/s, *P* < 0.001; Table 1). No correlation between age and SPV_max_ was observed for either sex (r = –0.08, *P* = 0.31 for both sexes; r = –0.04, *P* = 0.74 for males; r = –0.09, *P* = 0.4 for females; Fig. 2A). For vHIT, the mean Gain_60 ms_ across 96 patients was 0.94 ± 0.23 and not significantly different between males (0.98 ± 0.16) and females (0.90 ± 0.27; Table 1). No correlation between age and Gain_60 ms_ was observed for either sex (r = –0.11, *P* = 0.27 for both sexes; r = –0.004, *P* = 0.98 for males; r = –0.19, *P* = 0.18 for females; Fig. 2B). To confirm that both vestibular assessments both represent HSC function and are, therefore, correlated to each other, we performed a correlation analysis of SPV_max_ and Gain_60ms_, which showed the expected significant, positive correlation (r = 0.38, *P* = 0.002).

### Association Between Age-related Hearing Loss and Vestibular Hypofunction

Although no significant correlation between age and HSC function was observed when examining outcome measures from either caloric reflex testing or vHIT, we hypothesized that HSC might nevertheless be predicted by hearing function. To test this hypothesis, a linear multivariable regression analysis was performed to test whether HSC hypofunction might be predicted by high-frequency hearing loss. For both caloric reflex testing and vHIT outcome measures, three models were used. In the first model, age and sex were included as covariates. In the second model, the hFI was added as a covariate to investigate the effect of hFI on HSC function. In the third model, the interaction between the hFI and sex was tested, this interaction will further be referred to as “interaction term”. For caloric testing (Table 2), male sex negatively predicted SPV_max_ in both models 1 and 2. However, neither age (model 1) nor hFI (model 2) predicted SPV_max_. Model 3 shows that adding the interaction term did not change the independent association between SPV_max_ and sex. For vHIT (Table 3), neither sex nor age predicted Gain_60 ms_ in model 1. In model 2, male sex positively predicted Gain_60 ms_, but hFI did not predict Gain_60ms_, suggesting that the association with sex is mediated by hFI. Adding the interaction term in model 3, results in a nonsignificant association between Gain_60ms_ and sex; however, the association between Gain_60ms_ and the interaction term is also not significant. Since the univariate analysis revealed that the occurrence of diabetes mellitus was significantly different between males and females (*P* = 0.046), we performed additional regression analyses by including diabetes as a covariate to predict HSC function. We did not find significant independent effects of diabetes on the outcome measures of either caloric testing function or vHIT. Moreover, adding diabetes mellitus in the model did not change the effect sizes or significance levels of other covariates.

**TABLE 2.** Linear multivariable regression analysis to test whether sex, age, or hFI predict reduced peripheral vestibular function assessed by caloric testing

Model 1	Estimate	SE	Statistic	*P*	Model 2	Estimate	SE	Statistic	*P*	Model 3	Estimate	SE	Statistic	*P*
(Intercept)	62.477	8.923	7.002	0.000		64.467	9.284	6.944	0.000		67.448	9.456	7.133	0.000
Age	–0.128	0.140	–0.917	0.361		–0.202	0.168	–1.199	0.232		–0.175	0.169	–1.040	0.300
Sex (Male)	–12.138	3.547	–3.422	0.001^*^		–12.557	3.590	–3.497	0.001^*^		–21.419	6.856	–3.124	0.002^*^
hFI						0.113	0.144	0.787	0.432		–0.092	0.197	–0.469	0.640
											0.364	0.240	1.515	0.132

In both models 1, 2, and 3, SPV_max_ was used as the outcome variable. In model 1, age and sex were used as predictors. In model 2, hFI was additionally included as a predictor. In model 3, the interaction between hFI and sex was tested.

* Indicates significant associations.

hFI indicates high Fletcher index; SE, standard error.

**TABLE 3.** Linear multivariable regression analysis to test whether sex, age, or hFI predict reduced peripheral vestibular function assessed by vHIT

Model 1	Estimate	SE	Statistic	*P*	Model 2	Estimate	SE	Statistic	*P*	Model 3	Estimate	SE	Statistic	*P*
(Intercept)	1.032	0.113	9.123	0.000		0.990	0.115	8.579	0.000		1.009	0.118	8.555	0.000
Age	–0.002	0.002	–1.219	0.226		0.000	0.002	–0.207	0.836		0.000	0.002	–0.145	0.885
Sex (Male)	0.085	0.046	1.837	0.069		0.095	0.046	2.042	0.044^*^		0.032	0.088	0.365	0.716
hFI				–0.003	0.002	–1.595	0.114				–0.004	0.002	–1.754	0.083
Sex X hFI											0.003	0.003	0.829	0.409

In both models 1, 2, and 3, Gain_60 ms_ was used as the outcome variable. In model 1, age and sex were used as predictors. In model 2, hFI was additionally included as a predictor. In model 3, the interaction between hFI and sex was tested.

* Indicates significant associations.

hFI indicates high Fletcher index; SE, standard error; vHIT, video head-impulse test.

Since vHIT testing was implemented later in this clinic there might be concerns about selection bias in these analyses. We therefore repeated the analyses in only the patients that were both tested using the caloric reflex testing and vHIT to investigate this. This resulted in a considerable downsizing of the dataset to 88 patients. Correlations between age and hFI (r = 0.54, *P* < 0.001), SPV_max_ (r = 0.006, *P* = 0.95) and Gain_60ms_ (r = -0.094, *P* = 0.38) were comparable to the original dataset. Regression analysis for the caloric testing, did not identify sex or hFI to predict for SPV_max_ in models 1 and 2 (Table 4). The effect sizes are generally the same size and direction in these models. In model 3, the adding the interaction term show an almost significant (*P* = 0.058) association between SPV_max_ and sex. For vHIT testing, age and sex and hFI did not predict for Gain_60ms_ (Table 5). Adding the interaction term as a covariate in model 3 identifies a significant negative association between Gain_60ms_ and hFI. Again, effect sizes and directions were generally similar in the reduced dataset, compared with the complete dataset. In summary, in the smaller dataset, we were able to identify a significant negative association between Gain_60ms_ and hFI, this trend was observable in the complete dataset as well, but we did not observe a significant effect. Furthermore, we can draw similar conclusions from the analyses in the reduced data compared with the complete dataset, however, especially for the caloric testing data the power to detect significant effects is drastically reduced.

**TABLE 4.** Linear multivariable regression analysis to test whether sex, age, or hFI predict reduced peripheral vestibular function assessed by caloric testing in patients that were also assessed using vHIT

Model 1	Estimate	SE	Statistic	*P*	Model 2	Estimate	SE	Statistic	*P*	Model 3	Estimate	SE	Statistic	*P*
(Intercept)	48.344	10.855	4.454	0.000		47.600	11.262	4.227	0.000		50.588	11.454	4.417	0.000
Age	0.019	0.172	0.113	0.910		0.050	0.206	0.241	0.810		0.071	0.206	0.344	0.732
Sex (Male)	–7.135	4.450	–1.603	0.113		–7.010	4.499	–1.558	0.123		–16.379	8.534	––1.919	0.058
hFI						–0.049	0.183	–0.268	0.789		–0.244	0.237	–1.030	0.306
											0.397	0.308	1.290	0.201

In both models 1, 2 and 3, SPV_max_ was used as the outcome variable. In model 1, age and sex were used as predictors. In model 2, hFI was additionally included as a predictor. In model 3, the interaction between hFI and sex was tested.

^*^Indicates significant associations.

hFI indicates high Fletcher index; SE, standard error; vHIT, video head-impulse test.

**TABLE 5.** Linear multivariable regression analysis to test whether sex, age, or hFI predict reduced peripheral vestibular function assessed by vHIT in patients that were also assessed for caloric testing

Model 1	Estimate	SE	Statistic	*P*	Model 2	Estimate	SE	Statistic	*P*	Model 3	Estimate	SE	Statistic	*P*
(Intercept)	1.025	0.120	8.539	0.000		0.968	0.122	7.934	0.000		1.000	0.124	8.054	0.000
Age	–0.002	0.002	–0.938	0.351		0.001	0.002	0.236	0.814		0.001	0.002	0.337	0.737
Sex (Male)	0.074	0.049	1.505	0.136		0.084	0.049	1.717	0.090		–0.016	0.092	–0.172	0.864
hFI						–0.004	0.002	–1.903	0.061		–0.006	0.003	–2.278	0.025^*^
Sex X hFI											0.004	0.003	1.265	0.209

In both models 1, 2, and 3, Gain_60 ms_ was used as the outcome variable. In model 1, age and sex were used as predictors. In model 2, hFI was additionally included as a predictor. In model 3, the interaction between hFI and sex was tested.

* Indicates significant associations.

hFI indicates high Fletcher index; SE, standard error; vHIT, video head-impulse test.

## DISCUSSION

Our study investigated age-related changes in auditory and vestibular function using paired measurements of hearing and vestibular (HSC) function acquired from the healthy ear of patients undergoing the routine diagnostic workup for CPA tumors. This work revealed four main findings. First, we found a significant association between age and high-frequency hearing loss assessed using PTA (Fig. 1). These results are consistent with audiometric findings across much larger clinical cohorts (Parthasarathy et al. 2020) and suggest that the cohort investigated shows “normal” age-related high-frequency hearing loss in the healthy ear despite CPA tumor in the opposite ear.

Second, we found no association between age and vestibular (HSC) hypofunction assessed using either caloric reflex testing or vHIT (Fig. 2). These results are consistent with previous work using vHIT to assess semicircular canal function in 91 healthy subjects well-stratified over 8 decades between the ages of 10 and 89 years old (McGarvie et al. 2015). On the other hand, these results contradict previous studies motivating this work (Zuniga et al. 2012; Agrawal et al. 2012). Differences between findings may have resulted from the different age-range included and different test used to interrogate semicircular canal function (htDVA) in this previous work. However, our results also differ from another similarly sized study (132 subjects) that also assessed HSC function using vHIT (Treviño-González et al. 2021). Important to note, however, is that this work reported only a modest but significant decrease in Gain_60 ms_ in the left (but not right) HC. We did not find significant differences in Gain_60 ms_ between the left and right (healthy) ears in this study (*P* = 0.45). Another study assessing age-related changes in HSC in 91 individuals using caloric reflex testing also reported significantly reduced SPV in the subgroup of individuals older than 60 years compared with the subgroup of younger subjects (Felipe & Cavazos 2021). We did not observe significant effects of age on SPV; however, we did not use age-stratified groups. It is important to note that, most previous work (i.e., McGarvie et al. 2015; Treviño-González et al. 2021; Felipe & Cavazos 2021) did not include audiometric assessments that would have allowed investigation of the association between age-related high-frequency hearing loss and vestibular hypofunction. In total, this work and the work of others, suggests that semicircular canal function is remarkably stable across ages despite the age-related morphological loss of sensorineural structures (Rosenhall & Rubin 1975; Walther & Westhofen 2007; Merchant et al. 2000; Zalewski 2015; Ji & Zhai 2018). These seemingly incongruous findings suggest that function is robust in the face of sensorineural loss and that other central mechanisms compensate for sensorineural loss. Future work correlating peripheral vestibular histopathological and functional changes within individuals, as has been done to correlate histopathological changes in the cochlea with age-related hearing loss (Wu et al. 2020), and better assessment of central compensation in response to reduced peripheral vestibular function (Tighilet et al. 2019), is necessary to understand the mechanisms involved in age-related vestibular hypofunction.

Third, we found that age-related high-frequency hearing loss did not predict HSC hypofunction. This finding is consistent with previous work motivating this study, in which the investigators reported no significant correlation between age-related high-frequency hearing loss and semicircular canal hypofunction (Zuniga et al. 2012). These findings might suggest that age-related hearing loss and semicircular canal hypofunction occur independently from one another. However, this previous work did report a significant correlation between age-related high-frequency hearing loss and saccular hypofunction. We did not assess saccular function in this study. Our findings also do not align with a recent study linking hearing loss with postural instability in the elderly (Bang et al. 2020). It is important to note that, postural stability reflects not only vestibular function but also proprioceptive function and skeletomuscular integrity, both of which also show age-related declines that may confound interpretation (Shaffer & Harrison 2007; Gomes et al. 2017). Altogether, these findings indicate that the patterns of age-related decline in function vary across the cochlea and five vestibular end-organs and that more comprehensive and integrated assessment of inner ear function and tests of balance and stability are needed to understand the complex interactions linking age-related hearing loss and reduced peripheral vestibular function with imbalance in the elderly.

Fourth, we found sex-specific differences in audiometric function and vestibular function assessed using caloric testing. When comparing age-related high-frequency hearing loss between men and women, men showed significantly worse age-related high-frequency hearing loss compared with women. This finding is not surprising and well documented (Hoffman et al. 2017). We also observed significantly reduced caloric reflex response in men compared with women but no significant difference in the age-related decline of the caloric response between sexes when analyzing results from caloric reflex testing. A previous study found no significant difference between sexes when probing HSC function with the caloric reflex test (Felipe & Cavazos 2021); however, the mean age of this group was younger (43 ± 15 years in this study compared with 62.1 ± 12.7 years in our study). In contrast, we found enhanced HSC function in males compared with women when probing HSC function using vHIT, consistent with previous findings (Mahfuz et al. 2021). These findings may reflect sex-specific differences in low and high-frequency vestibular responses. In general, sex differences in vestibular function and dysfunction are well recognized, with females showing greater prevalence and severity of vestibular dysfunction compared with males (Smith et al. 2019). Although tempting to speculate that these differences arise from hormonal differences, the effects persist long after menopause, suggesting more complex interactions are involved. Nonetheless, our results reinforce the importance of considering sex-specific differences and warrant consideration of obtaining sex-specific normative values for identifying age-related vestibular hypofunction.

This study has limitations that should be considered. Some of these limitations are shared across similar studies. Specifically, our assessments of vestibular function assess the VOR, which involves central circuits that may themselves show age-related changes and/or mask (via central compensation) peripheral vestibular deficits. In addition, due to the retrospective cross-sectional design of our study, some data are missing. Specifically, in this study, all three outcome measures from audiometric assessment, caloric reflex testing, and vHIT results were available for 88 out 185 patients. Moreover, the cross-sectional design of our study does not allow us to make inferences about causality. A limitation specific to this study is the patient population investigated. Although the medical histories of these patients showed the prevalence of major health comorbidities was low, patients with CPA tumors do not represent a truly healthy aging population (Early et al. 2020). Moreover, because of the CPA tumor, these patients have unilateral vestibular deficits. The normal VOR reflects the combined effect of excitation and inhibition from both ears. Thus, in patients, head rotations to the healthy side will lack inhibition and, therefore, eye velocity will not quite completely compensate for head velocity (Halmagyi et al. 2017). The extent of deviation depends on many factors, including the degree of vestibular deficit and central compensation, which likely vary among the patients in this cohort. In patients with surgically verified unilateral vestibular loss, there is a reduction in VOR gain for head rotations to the healthy side (Halmagyi et al. 1990). Thus, unilateral vestibular deficits in patients with CPA tumor might mask, to varying degrees, age-related vestibular hypofunction. Another limitation of this study was that there was no information on environmental factors, for example noise exposure history, that could negatively impact auditory function, and or medication use that could negatively influence vestibular function. As many know age-related hearing loss is mediated by environmental factors, including noise exposure and ototoxic exposure (Wong & Ryan 2015). Thus, our work highlights the need for prospective cohort studies that include vestibular end-organ assessments in healthy aging individuals.

In conclusion, our work did not find evidence indicating age-related decline in HSC function or an association between age-related high-frequency hearing loss and age-related decline in HSC function. Nonetheless, our study highlights the need for sex-specific normative values for identifying age-related reduced peripheral vestibular function. It is important to note that, the sense of balance involves the integration of multiple sensory systems, including the proprioception, visual, and vestibular system (Brandt & Dieterich 2017). Thus, this work also highlights the need for future work linking comprehensive assessments of inner ear function with tests of balance and stability to understand the complex interactions underlying hearing loss and imbalance, especially in the elderly.

## ACKNOWLEDGMENTS

This study was supported by funds from the Heinsius Houbolt Foundation to S.J.P. N.M.A.S. has been funded by an MD/PhD scholarship (number 16–59) from the Junior Scientific Masterclass (Graduate School of Medical Sciences, University of Groningen, University Medical Center Groningen, Groningen, Netherlands).
